# Cardiometabolic Comorbidities in Psoriasis and Psoriatic Arthritis

**DOI:** 10.3390/ijms19010058

**Published:** 2017-12-25

**Authors:** Lluís Puig

**Affiliations:** Department of Dermatology, Hospital de la Santa Creu i Sant Pau, Universitat Autònoma de Barcelona, Sant Quintí 89, 08041 Barcelona, Spain; lpuig@santpau.cat

**Keywords:** psoriasis, psoriatic arthritis, inflammation, cardiovascular disease, cardiovascular risk, metabolic syndrome, atherosclerosis

## Abstract

There is solid epidemiologic evidence linking psoriasis and psoriatic arthritis (PsA) to cardiovascular risk factors and an increased risk of developing cardiovascular disease. Chronic inflammation, with shared pathways and cytokines common to metabolic syndrome, atherosclerosis and psoriasis, might provide the basis for the cardiovascular and metabolic comorbidities of psoriasis and PsA. The purpose of this manuscript is to review recent evidence about the epidemiology and underlying mechanisms of cardiovascular risk factors and cardiovascular disease in patients with psoriasis and/or PsA; the use of analytical determinations, physiologic measures and imaging techniques as surrogate biomarkers of atherosclerosis, endothelial dysfunction and cardiovascular disease in these patients; and the epidemiological and clinical data, including results of clinical trials, supporting a cardioprotective role of anti-inflammatory and disease-modifying treatment in psoriasis and PsA.

## 1. Introduction

Psoriasis is a common immune-mediated chronic inflammatory skin disease that is characterized by the altered proliferation and differentiation of keratinocytes and skin inflammation with participation of both the innate and adaptive immune systems, being primarily driven by pathogenic T cells that produce high levels of interleukin 17 (IL-17) in response to IL-23. The central pathophysiologic role of the IL-23/IL-17A axis in psoriasis has been confirmed by the therapeutic success with targeted monoclonal antibodies; the effect of tumor necrosis factor α (TNF) antagonists is probably exerted indirectly, since TNF is an upstream inducer of IL-23 and acts synergistically with IL-17 increasing the up-regulation of many psoriasis-related pro-inflammatory genes in keratinocytes [1]. TNF and other inflammatory mediators [2] have been postulated to maintain a state of chronic systemic inflammation that would induce insulin resistance, endothelial dysfunction, and cardiovascular diseases [3], together with an increasing number of comorbidities, including metabolic syndrome (obesity, hypertension, dyslipidemia, and diabetes), chronic kidney disease, gastrointestinal disease, mood disorders, and malignancy [4].

While Genome-Wide Association Studies (GWAS) have shown a large overlap of genes that govern coronary disease and metabolic syndrome, the genetics of psoriasis and these comorbidities are mostly independent [5,6]. Thus, in contrast to psoriatic arthritis (PsA) [7] and Crohn’s disease [8], which share genetically based pathomechanisms with psoriasis, chronic inflammation would provide the basis for the cardiovascular and metabolic comorbidities of psoriasis. On the other hand, a network approach has demonstrated a certain degree of commonality between psoriasis and some comorbidities as regards genes/proteins, biological processes and pathways; type 2 diabetes mellitus led the molecular comorbidity index followed by rheumatoid arthritis, Alzheimer’s disease, myocardial infarction and obesity [9]. Rather than genetic associations, an unfavorable lifestyle (smoking, obesity, no regular physical activity, and unhealthy diet), perhaps associated with the disease-associated inflammatory burden or in patients with psoriasis and/or PsA, might contribute to cardiovascular comorbidity in these conditions.

This paper will focus on cardiovascular comorbidities of psoriasis, with an overview of the epidemiological evidence linking psoriasis and PsA to cardiovascular diseases and risk factors, the inflammatory pathways common to metabolic syndrome, atherosclerosis and psoriasis, the serum biomarkers and imaging techniques used to assess subclinical atherosclerosis in patients with psoriasis and PsA, and the epidemiological and initial clinical data suggesting a therapeutic effect of the anti-inflammatory approach in preventing cardiovascular disease or its surrogate markers in patients with psoriasis.

## 2. Cardiovascular Comorbidities of Psoriasis and Psoriatic Arthritis

### 2.1. Cardiovascular Diseases

In the last decade, modern epidemiological studies have demonstrated that psoriasis, particularly severe disease (usually defined by requirement of phototherapy or systemic treatment), is associated with increased mortality [10] and medical comorbidity [11]. The pioneering study by Gelfand et al. in 2006 [12] provided strong evidence suggesting that psoriasis (especially severe psoriasis) is an independent risk factor for myocardial infarction, especially in young patients. This study used prospective data (collected between 1987 and 2002) from the United Kingdom General Practice Research Database, and included approximately 127,000 patients with mild psoriasis, 3800 patients with severe psoriasis, and 500,000 control patients. The adjusted (taking into account risk factors including hypertension, diabetes, history of previous myocardial infarction, hyperlipidemia, cigarette smoking, age, sex, and body mass index) relative risk for myocardial infarction was 1.29 (95% confidence interval CI, 1.14–1.46) for a 30-year-old patient with mild psoriasis and 3.10 (95% CI, 1.98–4.86) if psoriasis was severe (defined by the need for systemic treatment). The adjusted relative risk (RR) remained elevated, albeit less so, in older age groups (up to the age of 70). For a 60-year-old patient with mild or severe psoriasis, the adjusted RR of having a myocardial infarction is 1.08 (95% CI, 1.03–1.13) and 1.36 (95% CI, 1.13–1.64), respectively, but the increased frequency of myocardial infarction in older patient populations accounts for a much larger absolute risk than in younger patients. Interestingly, the prognosis after acute myocardial infarction seems to be worse in patients with psoriasis [13]. Furthermore, patients with severe psoriasis have an increased risk of cardiovascular death [14] that appears to be independent of traditional cardiovascular risk factors [15].

Subsequently, numerous epidemiologic studies and several systematic reviews and meta-analyses have suggested, with some exceptions, that psoriasis is an independent risk factor for myocardial infarction, stroke, and death caused by cardiovascular disease, collectively termed major adverse cardiovascular events (MACE), as reviewed by Takeshita et al. [16] The risks of myocardial infarction, stroke, and cardiovascular mortality appear to be greatest among those with severe disease, but still significantly increased in patients with mild psoriasis with respect to controls [17,18]. Longer duration of disease has also been associated with increased risk of cardiovascular disease: the risk of MACE increased 1.0% per additional year of psoriasis duration (HR 1.010; 95%CI 1.007–1.013) [19].

The prevalence of hypertension, obesity, hyperlipidemia, diabetes mellitus, and at least one cardiovascular event in PsA patients is significantly higher than in patients with psoriasis without arthritis, with unadjusted odds ratios (ORs) ranging from 1.54 to 2.59 [20]. Interestingly, in a cohort study in which neither psoriasis nor severe psoriasis were associated with an increased risk of major cardiovascular events over 3–5 years, after adjusting for known cardiovascular disease risk factors [21], the risk of a major cardiovascular event was 36% higher in patients with psoriasis who also had inflammatory arthritis compared with those who did not.

### 2.2. Cardiovascular Risk Factors and Metabolic Syndrome

The prevalence of traditional cardiovascular risk factors such as obesity, hypertension, diabetes, dyslipidemia, metabolic syndrome and smoking is increased in psoriasis [16,22].

The association between psoriasis and obesity has been well established, as have the implications of obesity regarding treatment of psoriasis [23]. The odds ratio (OR) for the association between psoriasis and obesity by body mass index is 1.8 (95% CI 1.4–2.2) [24]. When severity is taken into account, the pooled OR for obesity was 1.46 (95% CI 1.17–1.82) among patients with mild psoriasis and 2.23 (95% CI 1.63–3.05) for those with severe psoriasis [25]. Obesity is recognized as an independent risk factor for psoriasis [26]: obesity and high abdominal fat mass doubles the risk of psoriasis, and long-term weight gain substantially increases psoriasis risk [27].

A meta-analysis of 24 observational studies found a pooled OR for the association between psoriasis and hypertension to be 1.58 (95% CI, 1.42–1.76) [28]. The OR for hypertension was 1.30 (95% CI 1.15–1.47) for patients with mild psoriasis and 1.49 (95% CI 1.20–1.86) for severe psoriasis [28]. In addition, the likelihood of poorly controlled hypertension appears to increase with more severe skin disease, independent of body mass index (BMI) and other risk factors [29].

The pooled OR of psoriasis associated with diabetes in a meta-analysis of 44 observational studies was 1.76 (95% CI 1.59–1.96). Patients with PsA had the highest OR (2.18, 95% CI 1.36–3.50) [30]. Patients with severe psoriasis also had a higher OR (2.10, 95% CI 1.73–2.55). Moreover, diabetic patients with psoriasis appear to be more likely to suffer from micro- and macrovascular diabetes complications than diabetic patients without psoriasis [31].

In a systematic review, 20 of 25 included studies found significant associations between psoriasis and dyslipidemia, with ORs ranging from 1.04 to 5.55 [32]. In studies that took into account the severity of psoriasis, higher odds of dyslipidemia were seen in patients with severe psoriasis (range, 1.36 to 5.55) than in patients with mild psoriasis (range, 1.10 to 3.38) [32].

According to a recently published meta-analysis of 35 observational studies from 20 countries with 1,450,188 total participants, of which 46,714 were psoriasis patients, the pooled OR based on random effects analysis was 2.14 (95% CI 1.84 ± 2.48) [33]. In a cross-sectional study in the United Kingdom, the prevalence of metabolic syndrome correlated directly with body surface area (BSA) affected by psoriasis, varying in a “dose-response” manner, from mild (≤2% BSA; adjusted OR 1.22, 95% CI 1.11–1.35) to severe psoriasis (>10% BSA; adjusted OR 1.98, 95% CI 1.62–2.43) [34].

Smoking has been found to be significantly associated with psoriasis, with a RR of 1.88 (95% CI, 1.66–2.13); in most publications, smoking is also associated with an increased severity of psoriasis [35]. Smoking has also been associated with an increased risk of incident psoriasis, with a possible dose-effect relationship [36].

The hazard ratio for depression in psoriasis is approximately 1.4–1.5 and increases with disease severity [37,38]. Depression is a risk factor for cardiovascular diseases, incident cardiovascular events, and mortality, and a diagnosis of depression at any time following coronary artery disease is associated with a two-fold higher risk of death [39]. Thus, the association of psoriasis with depression might be clinically relevant as regards cardiovascular disease and mortality. In patients with psoriasis, depression is associated with increased risk of myocardial infarction, stroke and cardiovascular death, especially during acute depression [40]. Depression may play an important role in promoting subclinical atherosclerosis beyond traditional cardiovascular risk factors, and even psoriasis itself as independent risk factor. In patients with psoriasis and self-reported depression, vascular inflammation—measured by 18-fluorodeoxyglucose positron emission tomography/computed tomography (FDG PET/CT)—and coronary plaque burden—measured by coronary CT angiography—have been found to be significantly increased, after adjustment for Framingham Risk Score, as compared to patients with psoriasis alone [41].

The cardiovascular burden may be higher in patients with PsA compared to those with psoriasis without arthritis [20]. The extent of atherosclerosis, measured by imaging modalities, is higher in PsA than in the general population and in patients with psoriasis alone [42]. The presence of arthritis may indicate an increased underlying systemic inflammation that may worsen comorbidities and cardiovascular outcomes. Obesity and its related metabolic abnormalities are more prevalent in patients with PsO and PsA than in those with other inflammatory arthritides [43,44,45]. Furthermore, obesity is associated with increased risk of incident PsA among PsO patients and in the general population [46].

In summary, the high prevalence of traditional cardiovascular risk factors and metabolic abnormalities contributes to the high cardiovascular burden in patients with psoriasis and PsA, and obesity but may also affect the risk of developing psoriasis and impact disease activity. The presence of systemic inflammation in combination with metabolic abnormalities may act in a synergistic manner to increase cardiovascular risk in these patients.

## 3. Metabolic Syndrome, Atherosclerosis and Psoriasis/Psoriatic Arthritis: Common Inflammatory Pathways

Excessive weight gain can provoke hypertension, type 2 diabetes, atherosclerosis and hyperlipidemia, which define the metabolic syndrome, but only half of obese patients with body mass index from 30 to 50 kg/m^2^ are metabolically unhealthy [47]. On the other hand, metabolic syndrome can transiently occur in lean individuals during infection, where increased secretion of TNF, IL-6 and IL-1β by macrophages induces a temporary insulin-resistant state [48]. While transient inflammation-mediated insulin resistance can be helpful, permanent inflammation makes it to become chronic; conversely, chronic inflammation of adipose tissue with infiltration of macrophages and T cells leads to insulin resistance, and eventually type 2 diabetes mellitus in obese individuals. Visceral obesity and insulin resistance are characterized by persistent production of abnormal adipocytokines such as TNF, IL-6, IL-1β, leptin, and adiponectin, which contribute to the development of a pro-inflammatory state and further a chronic, subclinical vascular inflammation which modulates and results in atherosclerotic processes [49]. The role of Th17-derived cytokines in the pathogenesis of obesity and related inflammatory diseases is increasingly recognized [50].

At the metabolic level, obesity is associated with elevated serum levels of free fatty acids, which sensitize human dendritic cells to amplify Th1/Th17 immune responses [51]; imiquimod-induced psoriasiform skin inflammation in obese mice is more severe than in lean mice, and is also associated with higher serum levels of IL-17, IL-22 and IL-23 [51]. Obesity has been shown to promote expansion of IL-17-producing T cells in adipose tissue (especially visceral fat) and peripheral tissues [52,53]. Accordingly, a significant increase in circulating IL-17 and IL-23 cytokines has also been observed in obese as compared with lean individuals, in humans [54]. Supporting the implication of IL-17 in the metabolic syndrome, the levels of IL-17R expression in liver or muscle have been shown to correlate with insulin resistance [55], and IL-17 blocking resulted in the decrease of hepatic inflammation in the non-alcoholic steatohepatitis syndrome [56].

Atherosclerosis, which was formerly viewed as a cholesterol storage disease leading to flow-limiting stenosis, is now defined as a chronic immune-mediated inflammatory disease that arises from a series of complex events that are triggered by endothelial dysfunction, lipid deposits in the arterial wall and infiltration of monocyte-derived macrophages. Several pro-inflammatory cytokines, especially IL-1β, TNF, and IL-6, are considered to be pathogenically relevant and susceptible to therapeutic intervention [57]. Again, the relevance of IL-17 in the process of atherogenesis is now becoming increasingly recognized [58]. Interestingly, using a bioinformatics approach of human skin and vascular tissue, IFN-γ and TNF have been determined to be the dominant pro-inflammatory signals linking atherosclerosis and psoriasis [59].

Apart from the involvement of common pro-inflammatory cytokines, the pathways underpinning the commonalities between the inflammation in psoriasis/PsA and atherosclerosis are far from clear. Comprehensive reviews of the genetic basis of coronary artery disease have recently been published [60,61], and associations have been found with genes involved in immune cell trafficking [62,63], inflammation, cell adhesion and transendothelial migration [64], as well as loci shared with Crohn’s disease and ulcerative colitis (probably disrupting the regulation by AP-1 transcription factor of the expression of SMAD3, a signal transducer in the transforming growth factor β pathway) [65], but the genetic association with psoriasis is largely unproven. 

Neutrophil-macrophage communication is involved in any inflammatory response, and specifically in the inflammation that aggravates atherosclerosis [66,67], and NETosis, or the ability of neutrophils to expel cytosolic and nuclear material forming extracellular traps that ensnare extracellular microbes, has been proposed to play a role in both atherosclerosis [66] and psoriasis [68], but this commonality seems far too general. 

## 4. Laboratory Biomarkers and Imaging of Subclinical Atherosclerosis

For epidemiologic purposes, the relative rarity of clinical endpoints such as MACE requires following large patient cohorts for extended periods of time. Therefore, serum biomarkers and surrogate markers such as carotid atherosclerosis, coronary calcifications, endothelial dysfunction and imaging techniques are commonly used for investigating the link between psoriasis, PsA, and cardiovascular disease. The currently available evidence has recently been the subject of an excellent review [69].

The clinical usefulness of C-reactive protein determinations in cardiovascular disease in the general population has been questioned among patients with pre-existing inflammatory conditions such as psoriasis [70]. High erythrocyte sedimentation rate values are associated with a higher burden of atherosclerosis and clinical cardiovascular events in patients with PsA [71,72]. In a study of serum levels cytokines and adhesion molecules involved in endothelial function, endothelin-1 has been found to correlate with erythrocyte sedimentation rate and Disease Activity Score 28 in PsA [73]. Increased levels of N-terminal pro B-type natriuretic peptide—a biomarker associated with an increased risk of cardiovascular death, myocardial infarction and heart failure—have been found in patients with psoriasis compared to controls [74], but the clinical relevance of this finding is as yet uncertain. Glyc A is a novel, recently validated assay that quantifies the nuclear magnetic resonance signal from *N*-acetylglucosamine and *N*-acetylgalactosamine moieties of plasma glycoproteins and has been proposed as an alternative to C-reactive protein (CRP) as a newer biomarker of systemic inflammation. Glyc A predicted subclinical atherosclerosis in the form of vascular inflammation by FDG-PET/CT, and coronary artery disease by coronary CT angiography in psoriasis [75].

Plasma asymmetric dymethylarginine (ADMA), a major endogenous inhibitor of nitric oxide synthetase, is a newly discovered risk factor for endothelial dysfunction in atherosclerosis and a predictor of cardiovascular risk [76]. In a study of 22 patients with PsA and 35 healthy controls with no history or current signs of cardiovascular disease, ADMA levels were significantly higher in PsA patients, who also had a significantly reduced coronary flow reserve (CFR) [77]. CFR—the ratio of hyperemic to resting diastolic flow velocity in the left anterior descending coronary artery measured by transthoracic echocardiography at rest and during adenosine infusion—is a highly sensitive diagnostic marker of coronary artery disease: a CFR of less than 2 accurately predicts the presence of severe (i.e., >70%) coronary stenosis [78]. In a study of 56 young patients with psoriasis (aged 37 ± 3 years, 42 males) without clinical evidence of cardiovascular diseases, and 56 controls matched for age and gender, CFR was lower than in controls, and abnormal (≤2.5) in 22% vs. 0% controls, *p* < 0.0001. At multivariable analysis Psoriasis Area and Severity Index (PASI) remained the only determinant of CFR ≤ 2.5 (*p* = 0.02) [79]. These results have been confirmed in another study with 36 patients with psoriasis and 56 healthy volunteers, in which CFR was significantly and inversely correlated with disease duration, PASI score, and high sensitivity CRP [80].

Endothelial dysfunction is considered the earliest phase of atherosclerosis and can be measured by flow-mediated dilation and pulse wave velocity. Significant impairment of flow-mediated dilation compared to healthy controls has been found in patients with psoriasis [81,82,83], and PsA [84,85]. Arterial stiffness, as measured by pulse wave velocity, is greater in patients with moderate to severe psoriasis and in patients with PsA compared to controls [86]; the preponderance of literature in a 2014 review suggests that endothelial function is significantly impaired in patients with psoriasis and PsA [87].

Carotid atherosclerosis can be measured ultrasonographically by carotid intima media thickness and total plaque area, which have been found to be increased compared to controls in patients with psoriasis [81,88] and PsA [89,90,91,92]. Patients with PsA have increased carotid total plaque area compared to patients with psoriasis, independent of traditional cardiovascular risk factors [42]. Interestingly, in a study of 411 patients with PsA and psoriasis without PsA, HLA-B*13:02 and HLA-C*06:02 alleles have been found to be associated with more severe atherosclerosis and higher values of erythrocyte sedimentation rate over time (suggesting a higher level of systemic inflammation) after adjusting for cardiovascular risk factors [93]. 

Coronary artery calcification, as detected by CT, has been found to be more prevalent in patients with psoriasis compared to controls [94,95,96,97]. Coronary artery calcium increase in patients with psoriasis is similar to that of patients with type 2 diabetes after adjustment for body mass index [98]. Excessive quantity of epicardial adipose tissue, a risk factor for coronary artery disease, has also been found to be increased in CT studies of psoriasis patients in comparison to controls [94,99].

Coronary CT angiography (CCTA) is used to investigate coronary atherosclerosis, enabling identification of rupture-prone plaques, and has been proposed as an independent predictor of cardiovascular events [100].

A recently published study sought to compare total coronary plaque burden and non-calcified coronary plaque burden and high-risk plaque prevalence between patients with psoriasis (*n* = 105), patients with hyperlipidemia eligible for statin therapy (*n* = 100), and healthy volunteers without psoriasis (*n* = 25) [101]. A consecutive sample of the first 50 patients with psoriasis was scanned again one year after therapy. Despite being younger and at lower traditional risk than patients with hyperlipidemia, patients with psoriasis had increased non-calcified coronary plaque burden and similar high-risk plaque prevalence. In comparison to healthy volunteers, patients with psoriasis had increased total coronary plaque burden, non-calcified coronary plaque burden and high-risk plaque prevalence beyond traditional risk (OR 6.0; 95% CI 1.1–31.7). Last, improvement in psoriasis severity among patients with psoriasis followed for one year was associated with improvement in total coronary plaque burden beyond traditional risk factors.

A recent study assessing 90 PsA patients matched to controls found that PsA was an independent risk factor for increased prevalence, burden and severity of coronary atherosclerosis. Longer duration of PsA, older age and male gender were associated with higher risk of having coronary plaques. As compared to controls, in patients with PsA the risk of finding unstable non-calcified plaques was double, the risk of obstructive coronary plaques was four-fold, and the risk of three-vessel coronary disease was increased by a factor of 10 [102].

FDG-PET/CT is a novel, validated technique to measure in vivo whole-body inflammation, including high sensitivity for macrophage activity in the early, subclinical inflammation of atherosclerosis [103]. FDG is taken up by cells in proportion to their metabolic activity and quantifies vascular inflammation as a standardized uptake value. The measurement of vascular inflammation by FDG-PET/CT has evolved as an acceptable surrogate inflammatory marker that can be modulated with therapy and may prognosticate stroke and myocardial infarction [104].

In a pilot study of six patients with moderate to severe psoriasis versus controls, FDG-PET/CT demonstrated increased metabolic activity in the liver, increased clinical and subclinical joint inflammation, and increased aortic inflammation even after adjustment for cardiovascular risk factors. Inflammation observed in the aorta suggested that aortas of patients with psoriasis were aged ten years compared to their age-matched control cohorts [105].

In a FDG-PET/CT study performed in adult patients with mild to moderate psoriasis (*n* = 60, mean age 47 years, median PASI 5.4) and controls (*n* = 20, mean age 41 years), all of them with low cardiovascular risk, increasing PASI was associated with an increase in the aortic target-to-background ratio [106]. This association changed little after adjustment for age, sex, and Framingham risk score.

In a study of 190 patients with psoriasis who were young and had low cardiovascular risk by traditional risk scores but a high prevalence of cardiometabolic diseases, vascular inflammation by FDG-PET/CT has been found to be significantly associated with disease duration [19].

As regards PsA, in a FDG-PET/CT study on 38 patients with psoriasis and 27 patients with PsA, vascular inflammation was greater in patients with sacroiliitis defined by CT compared to those without sacroiliitis [107]. There were associations between PsA and aortic inflammation and between sacroiliitis and aortic inflammation after adjusting for cardiovascular disease risk factors. Sacroiliitis predicted vascular inflammation beyond PsA and cardiovascular risk factors.

## 5. Therapeutic Intervention

### 5.1. Epidemiologic Evidence of Anti-Inflammatory Interventions on Cardiovascular Outcomes in Psoriasis/Psoriatic Arthritis (PsA)

The first step in primary prevention of cardiovascular disease is to stratify patients according to their estimated cardiovascular risk using validated risk algorithms, which may underestimate the risk in patients with psoriasis and psoriatic arthritis [69]. Based on evidence established mainly in rheumatoid arthritis, EULAR has developed recommendations for cardiovascular risk management that increase (1.5×) the risk score based on traditional factors in patients with inflammatory arthritis that encompass PsA [108]. Several publications have found major gaps in the management of cardiovascular risk in psoriatic patients [109,110,111]. In one of these studies, 19%, 22% and 39% of psoriasis patients had undiagnosed diabetes mellitus, hypertension or hypercholesterolemia, respectively, and that up to 60% of them failed to achieve treatment targets for their cardiovascular risk factors [109]. When primary care physicians reported their screening practices for patients with psoriasis, only 43%, 11%, 27% and 30% of physicians screened for hypertension, dyslipidemia, diabetes mellitus and obesity, respectively [112].

Given the strong link between psoriasis/PsA, systemic inflammation, metabolic syndrome and cardiovascular events, control of the inflammation associated with psoriasis/PsA may apply beyond the skin and the joint disease to the prevention of cardiovascular diseases. Limited data exist about the impact of non-biologic disease-modifying anti-rheumatic drugs (DMARDs) on cardiovascular outcomes.

An association of methotrexate treatment with reduced cardiovascular risk has been found among patients with PsO in several population-based studies [113,114,115]. PsA patients using DMARDs have been reported to have a lower cardiovascular risk than those who were not using them [116]. A meta-analysis of ten cohort studies including patients with rheumatoid arthritis, psoriasis and PsA found that methotrexate therapy was associated with a 21% reduction in overall cardiovascular risk and 18% reduction in myocardial infarction risk [117].

The impact of TNF inhibitors on clinical cardiovascular events has been investigated in several observational studies, reviewed by Armstrong et al. in 2014 [118]. A retrospective cohort study in California found a 55% decrease in the risk of myocardial infarction in patients with psoriasis treated with TNF inhibitors compared to those treated with topical medications, and a 21% decrease when compared to phototherapy and oral agents, suggesting that TNF inhibition might be cardioprotective [119]. This is consistent with their potential effect on the metabolic syndrome, as illustrated by a retrospective study of consecutive patients with PsA treated with etanercept, adalimumab and methotrexate (70 with each treatment) and followed up for two years: an improvement in metabolic syndrome components was shown in patients treated with TNF inhibitors as compared to the methotrexate group [120].

The latest published meta-analysis on the effect of TNF inhibitors on cardiovascular events has included 49,795 patients with psoriasis and/or PsA from five studies [121]. TNF inhibitors were associated with a significant lower risk of cardiovascular events compared with topical treatment/phototherapy (RR, 0.58; 95% CI, 0.43 to 0.77) and methotrexate treatment (RR, 0.67; 95% CI, 0.52 to 0.88) in patients with psoriasis. Specifically, TNF inhibitors were linked to reduced incidence of myocardial infarction compared with topical/photo or methotrexate treatment (RR, 0.73; 95% CI, 0.59 to 0.90; and RR, 0.65; 95% CI, 0.48 to 0.89; respectively).

### 5.2. Clinical Evidence

There is limited evidence available on the effect of anti-inflammatory therapies on cardiovascular disease in psoriasis and/or PsA, but some large-scale studies have provided valuable information that might be applied to patients with these diseases. On the other hand, the sample size required to study the effect of anti-inflammatory interventions on biomarkers/imaging of atherosclerosis is smaller, and the available studies in patients with psoriasis and/or PsA are described in a subsequent section.

#### 5.2.1. Effect of Anti-Inflammatory Interventions on Cardiovascular Outcomes

Statins, beyond their effect on dyslipidemia, have been shown to reduce atherosclerotic plaque inflammation in patients at risk, especially in the presence of advanced coronary plaques [122], but the discussion of their therapeutic role exceeds the scope of this manuscript, and this potential effect has not yet been studied in detail in patients with psoriasis [123].

Two large, major trials aiming to assess the effect of anti-inflammatory treatment on prospective cardiovascular outcomes (secondary prevention) have been started: the Cardiovascular Inflammation Reduction Trial (CIRT) and the Canakinumab Anti-Inflammatory Thrombosis Outcomes Study (CANTOS).

The CIRT will randomize up to 7000 participants with cardiovascular disease and no systemic rheumatic disease to either low dose methotrexate (target dose: 15–20 mg/week) or placebo for an average follow-up period of 3–5 years; the primary endpoints of CIRT include recurrent cardiovascular events, incident diabetes, and all-cause mortality [124].

The CANTOS trial [125], involving 10,061 patients with previous myocardial infarction and a high-sensitivity C-reactive protein level ≥2 mg/L, has shown that treatment with the interleukin-1β antagonist canakinumab at a dose of 150 mg (but not 300 mg) every three months led to a significantly lower rate of recurrent cardiovascular events than placebo, independent of any effect on lipid levels. One unexpected finding from the CANTOS trial, the reduction on incident lung cancer in patients receiving canakinumab, suggests the possibility that the inflammatory pathway shared by comorbidities beyond cardiometabolic diseases might be influenced by therapeutic interventions.

#### 5.2.2. Effect of Anti-Inflammatory Interventions on Biomarkers/Imaging of Atherosclerosis

More information is available regarding the effects of biologic treatment on functional or imaging biomarkers of atherosclerosis. Most of the published evidence corresponds to TNF antagonists, but trials with biologics of other classes, such as secukinumab, have just been completed [126].

CFR in the left anterior descending coronary artery was measured by transthoracic Doppler echocardiography, at rest and during adenosine infusion, in a prospective study of 37 consecutive psoriasis patients (31 male; age, 37.7 ± 8.5 years) without cardiovascular disease, before and after an average of 6.3 months (range 5.5–7.1 months) of anti-TNF treatment [127]. Overall, CFR increased from 2.2 ± 0.7 to 3.02 ± 0.8 (*p* < 0.0001) after TNF inhibitors therapy, and the increase was correlated with high sensitivity CRP and TNF reduction (*p* = 0.004 and *p* = 0.02, respectively), but not with change in PASI (*p* = 0.5). These findings suggest that specific anti-inflammatory treatment may positively affect the coronary microvascular function.

Most studies included in a 2014 meta-analysis [87] showed that TNF inhibitors improve physiologic measures of endothelial function in psoriasis and psoriatic arthritis, but the evidence is somewhat contradictory, especially as regards the long-term effects of treatment (Table 1). In a study with 14 psoriasis patients, treatment with adalimumab for 12 weeks improved flow mediated dilation but had no effect on carotid-femoral pulse wave velocity measurements [128]. In a six-month prospective study, 29 patients with moderate to severe psoriasis exhibited improvement of endothelial function and arterial stiffness following anti-TNF therapy with adalimumab [129].

Several studies have investigated the effect of anti-TNF agents on endothelial cell function in patients with PsA. A two-year pilot study showed that treatment with anti-TNF agents may determine a reduction in carotid intima media thickness in PsA patients, associated with improvement in inflammatory markers, but independent of changes in lipid profiles [130]. In a study involving 224 patients with PsA (120 on TNF blockers and 104 on DMARDs), the carotid intima media thickness in PsA patients without cardiovascular risk factors was higher than in controls. Treatment duration inversely predicted carotid intima media thickness in PsA subjects on TNF blockers but not in those on DMARDs. Furthermore, carotid plaques were detected in 15.8% of PsA patients on TNF blockers and in 40.4% of those on DMARDs (*p* < 0.0001) [131]. Contradictory evidence was derived from another study in 32 patients with PsA who were treated with anti-TNF agents for two years and developed progression of carotid intima media thickness with no recovery of post-occlusion flow-mediated dilation of the brachial artery despite improvement in clinical status [132].

Several studies have included patients with PsA and other inflammatory arthropathies. Mazzoccoli et al. studied the flow-mediated dilatation and the carotid intima media thickness in 36 patients (11 with PsA and 25 with rheumatoid arthritis) after 8–12 weeks of treatment with etanercept (10), infliximab (13) and DMARDs (13). No statistically significant difference between the three groups was found for the ultrasonographic evaluation, but flow-mediated dilatation was shown to increase significantly after treatment with etanercept and infliximab [133]. In another study of 17 patients with rheumatoid arthritis, ankylosing spondylitis or PsA who had been treated with infliximab for at least 12 months, aortic pulse wave velocity was not found to change during the period between two infusions [134]. The same authors followed 55 patients with rheumatoid arthritis, ankylosing spondylitis or PsA with regular assessments of aortic stiffness (aortic pulse wave velocity), carotid intima media thickness, and plasma calprotectin for one year. Thirty-six patients starting with anti-TNF therapy were compared with a non-treatment group of 19 patients [135]. After one year, aortic pulse wave velocity was improved and carotid intima media thickness progression was reduced in the treatment group, but not in the control group. The significance of the association was retained in multivariable analyses, which also showed a longitudinal association of calprotectin with aortic pulse wave velocity. Interestingly, the same authors observed an improvement in aortic stiffness with anti-TNF therapy after only three months of treatment [136].

In a subset of 50 patients with psoriasis from a previously cited study [101] who were followed for one year, improvement in psoriasis severity (PASI), regardless of treatment, was associated with improvement in CCTA-assessed total coronary plaque burden and non-calcified coronary plaque burden beyond traditional risk factors.

Recent studies have used FDG-PET/CT to assess aortic vascular inflammation following treatment. In a total of 115 consecutively recruited patients with psoriasis who were followed for one year, psoriasis skin disease severity was associated with improvement in aortic vascular inflammation (assessed as target-to-background ratio) beyond traditional cardiovascular risk factors [137]. The mean (SD) age of the study population at one-year follow-up was 50.8 (12.8) years and 68 were men (59%), with low cardiovascular risk by Framingham risk score and mild-to-moderate psoriasis, with a median PASI score of 5.2 (interquartile range, 3.0–8.9). At follow-up, the total cohort had a median improvement in PASI score of 33%, with use of topical therapy (60%), biological therapy (66%, mostly anti-tumor necrosis factor) and phototherapy (15%). The improvement in aortic vascular inflammation was greater in those patients who achieved PASI75 response or better, and the association was strongest in those initiated with anti-TNF agents.

In another study, vascular inflammation was also studied using FDG PET/CT scans at baseline and after one year in PsA patients on TNF inhibitors (*n* = 21) and age and sex matched PsA patients not on any biologics (*n* = 13) [138]. Vascular inflammation was measured as target-to-background ratio, and statistical analyses included multivariable regression analysis adjusting for cardiovascular risk factors and statins. After one year, patients on TNF inhibitors had a reduction in target-to-background ratio (*p* = 0.03), independent of cardiovascular risk factors, which was not observed in those on other treatments [136].

These results are in contrast with those of another study, in which a total of 107 patients with psoriasis were randomized (1:1) to receive adalimumab for 52 weeks or placebo for 16 weeks followed by adalimumab for 52 weeks, and vascular inflammation was also assessed with FDG-PET/CT. The change from baseline in vessel wall target-to-background ratio from the ascending aorta (primary endpoint) and the carotids were not shown to differ between both groups at Week 16. After 52 weeks of treatment with adalimumab, the target-to-background ratio did not change significantly from baseline in the ascending aorta, but there was a modest increase in the carotids [139]. Perhaps some of the differences in results between these studies might be accounted on the choice of endpoints [140].

## 6. Conclusions

There is abundant evidence of the association between psoriasis/PsA, chronic inflammation and cardiovascular disease, but further research and population studies are required to better understand the pathomechanisms underpinning this association and to improve primary prevention, currently hampered by underdiagnosis and undertreatment of cardiovascular risk factors in these patients. Laboratory biomarkers and vascular imaging modalities might improve risk stratification algorithms and can be used as surrogate outcomes in therapeutic clinical trials, which are urgently required to establish the role of anti-inflammatory treatment modalities in preventing and reducing atherosclerotic plaque inflammation and coronary microvascular function.

There is a strong need to improve primary and secondary prevention of cardiovascular disease in patients with psoriasis and psoriatic arthritis. The components of metabolic syndrome should be adequately diagnosed; lifestyle changes should be actively encouraged; risk stratification should be adjusted in patients with psoriasis and PsA; and the adequate pharmaceutical interventions should be implemented, with adequate monitoring of its effectiveness. The specialists caring for patients with psoriasis and/or PsA should play an active role to achieve these goals in collaboration with general practitioners and cardiologists. Although there is some evidence that some drugs, and especially TNF inhibitors aimed to reduce the inflammatory burden in patients with severe psoriasis and active PsA, might be beneficial in reducing the risk of cardiovascular disease, this should not be their primary role based on the currently available data. On the other hand, safety considerations must be taken into account, and, even though some biologics have been associated with an increased risk of infection, none of them has proven to have a deleterious effect on cardiovascular risk factors or diseases, and they should be preferred to ciclosporin (which may worsen hypertension, dyslipidemia, diabetes or renal disease) or acitretin (which can induce or worsen dyslipidemia) in patients at risk.

## Figures and Tables

**Table 1:** TNF inhibitors and endothelial function in patients with psoriasis and PsA.

First Author	Year	Disease	Number of Patients Treated with TNF-Inhibitors	Duration of Treatment between Assessments	Drug	Improvement of Flow Mediated Dilatation	Improvement in Pulse Wave Velocity	Impaired Progression of Intima Media Thickness
Avgerinou [128]	2011	Psoriasis	14	12 weeks	ADA	YES	NO	NA
Pina [129]	2016	Psoriasis	29	6 months	ADA	YES	YES	NA
Tam [130]	2011	PsA	11	3 months	TNF inhibitors	NA	NA	YES
Tam [130]	2011	PsA	9	2 years	TNF inhibitors	NA	NA	NO
Di Minno [131]	2011	PsA	120	52, 24 months (mean, SD)	ADA, ETN, IFX	NA	NA	YES
Ramonda [132]	2014	PsA	32	2 years	ADA, ETN, IFX	NO	NA	NO
Mazzoccoli [133]	2010	PsA, RA	36	8–12 weeks	ETN, IFX	YES	NA	NO
Angel [134]	2011	PsA, RA, AS	17	8 weeks (previously treated for ≥1 year)	IFX	NA	NO	NA
Angel [135]	2012	PsA, RA, AS	36	1 year	ADA, ETN, IFX	NA	YES	YES
Angel [136]	2010	PsA, RA, AS	35	3 months	ADA, ETN, IFX	NA	YES	NA

ADA: adalimumab; AS: ankylosing spondylitis; ETN: etanercept; IFX: infliximab; NA: not assessed; PsA: psoriatic arthritis; RA: rheumatoid arthritis; TNF: tumor necrosis factor α.
